# Automated Classification of Pulmonary Nodules through a Retrospective Analysis of Conventional CT and Two-phase PET Images in Patients Undergoing Biopsy

**DOI:** 10.22038/AOJNMB.2018.12014

**Published:** 2019

**Authors:** Atsushi Teramoto, Masakazu Tsujimoto, Takahiro Inoue, Tetsuya Tsukamoto, Kazuyoshi Imaizumi, Hiroshi Toyama, Kuniaki Saito, Hiroshi Fujita

**Affiliations:** 1 Faculty of Radiological Technology, School of Health Sciences, Fujita Health University, Toyoake, Japan; 2 Fujita Health University Hospital, Toyoake, Japan; 3 School of Medicine, Fujita Health University, Toyoake, Japan; 4 Department of Electrical, Electronic, and Computer Engineering, Faculty of Engineering, Gifu University, Gifu, Japan

**Keywords:** Classification, Computer-aided diagnosis, Lung cancer, Pulmonary nodule, PET/CT

## Abstract

**Objective(s):**

Positron emission tomography/computed tomography (PET/CT) examination is commonly used for the evaluation of pulmonary nodules since it provides both anatomical and functional information. However, given the dependence of this evaluation on physician’s subjective judgment, the results could be variable. The purpose of this study was to develop an automated scheme for the classification of pulmonary nodules using early and delayed phase PET/CT and conventional CT images.

**Methods:**

We analysed 36 early and delayed phase PET/CT images in patients who underwent both PET/CT scan and lung biopsy, following bronchoscopy. In addition, conventional CT images at maximal inspiration were analysed. The images consisted of 18 malignant and 18 benign nodules. For the classification scheme, 25 types of shape and functional features were first calculated from the images. The random forest algorithm, which is a machine learning technique, was used for classification.

**Results:**

The evaluation of the characteristic features and classification accuracy was accomplished using collected images. There was a significant difference between the characteristic features of benign and malignant nodules with regard to standardised uptake value and texture. In terms of classification performance, 94.4% of the malignant nodules were identified correctly assuming that 72.2% of the benign nodules were diagnosed accurately. The accuracy rate of benign nodule detection by means of CT plus two-phase PET images was 44.4% and 11.1% higher than those obtained by CT images alone and CT plus early phase PET images, respectively.

**Conclusion:**

Based on the findings, the proposed method may be useful to improve the accuracy of malignancy analysis.

## Introduction

Lung cancer is a leading cause of cancer mortality among men and women. This disease is a serious public health problem in many countries (1). When a lung nodule is found during cancer screening, it is important to accurately classify the lesion as benign or malignant in order to institute appropriate therapy and improve the survival rate. 

Computed tomography (CT) is often used for lung cancer screening (2). According to the results of a national lung screening trial carried out in the United States (3), screening with low-dose CT scans reduced lung cancer mortality by 20%. Therefore, CT is regarded as a suitable diagnostic tool for the early detection of lung cancer. If a suspicious lesion is found by CT examination, positron emission tomography (PET)/CT examination is performed for detailed analysis. In this combined technique, PET images provide functional information while CT images render anatomical information, thereby facilitating a comprehensive analysis of the malignancy of nodules.

However, fluorodeoxyglucose (FDG) PET images of benign nodules, such as those associated with inflammatory diseases, often exhibit high uptake values similar to the images of malignant nodules; furthermore, their anatomical structures are similar. Therefore, it is often difficult to differentiate between benign and malignant nodules (4). In such cases, a bronchoscopic biopsy is performed; however, the procedure is invasive, and the patient faces great physical hardship.

In these cases, if the CT and PET images can be analysed in detail to quantify the degree of malignancy of the nodules, the need for excessive biopsy with its accompanying physical hardship can be reduced. With his background in mind, the present study was focused on the automated analysis of the malignant potential of the pulmonary nodule using PET/CT images.

Many studies have investigated the benign/malignant differentiation of pulmonary nodules by image analysis (5-10). For instance, Armato et al. proposed the automated analysis of pulmonary nodule using linear discriminant analysis with characteristic features obtained from CT images (5). The evaluation of 470 CT scans in a study revealed that the area under the receiver operating characteristic (ROC) curve was 0.79. In addition, Shen et al. (9) introduced a deep learning model of the multi-crop convolutional neural network to classify pulmonary nodules. The authors used 880 benign nodules and 495 malignant nodules from the lung image database consortium and image database resource initiative (LIDC/IDRI) dataset and obtained an accuracy of 87.14% with the model. 

Nie et al. developed a semi-automated scheme for distinguishing between benign and malignant pulmonary nodules by integrating PET and CT information. They evaluated three computer-aided diagnosis schemes based on an artificial neural network to distinguish between benign and pulmonary nodules using clinical information and image features. They reported that the combined use of PET and CT rendered a higher diagnostic accuracy, compared to the employment of CT alone or PET alone (10). 

However, to the best of our knowledge, the automated calculation of the characteristic values of pulmonary nodules based on PET and CT images have not been developed. The automated classification of pulmonary nodules can have a great practical value. Regarding this, the present study involved the proposition of an automated classification scheme of the pulmonary nodule using CT and PET images. The major objective of our study was to develop the characteristic features using both CT and PET images. Furthermore, we also developed a classification method using random forest, which is a kind of ensemble machine learning technique.

In this paper, first, the architecture of the developed classification method is described. In addition, the effectiveness of the classification of pulmonary nodules as evaluated with the original CT and PET image database is discussed.

## Methods

This study was approved by the Institutional Review Board. Informed consent was obtained from all patients under the condition that all data were anonymized (No. HM17-002). The current study was conducted on 36 early and delayed phase PET/CT images obtained from patients with a suspected diagnosis of lung cancer. In addition, conventional CT images at maximal inspiration were analysed. The cases were chosen from those whose differential diagnosis was difficult with diagnostic imaging alone, and final diagnosis was made by bronchoscopy and biopsy analysis.

The PET/CT imaging studies were performed by means of Siemens True Point mCT (Siemens). Both images were obtained with a matrix size of 200×200 pixels (voxel size: 4.07×4.07×2.00 mm^3^, scan time: 2.0 min/table) with free breathing. Image reconstruction was performed using the 3D-OSEM reconstruction algorithm. 

In addition, point-spread function, time-of-flight correction (PSF+TOF), and attenuation correction were performed by CT images. The PET images were converted to the voxel size of the CT image after reconstruction. Early and delayed PET imaging was performed 60 min and 120 min after the administration of 3.7 MBq/kg of FDG, respectively. These PET and CT images were aligned automatically by the PET/CT scanner.

 The conventional CT imaging was performed using Aquilion ONE (Toshiba, Tokyo, Japan) with a matrix size of 512×512 pixels (voxel size: 0.625×0.625×0.500 mm^3^) with the lung kernel. In case the CT examination was carried out more than once, the images taken with the shortest interval from the PET/CT examination were selected.

Out of a total of 18 benign cases, 13 cases were finally diagnosed by biopsy, and the remaining 5 cases were confirmed to be benign by a follow-up examination of at least 3 years. Out of 25 malignant cases, 1, 4, and 13 cases were small cell carcinoma, squamous cell carcinomas and adenocarcinomas respectively. The mean ages of the patients in the malignant and benign nodule groups were 72.2±7.6 and 65.3±10.2 years, respectively.

**Figure 1:**
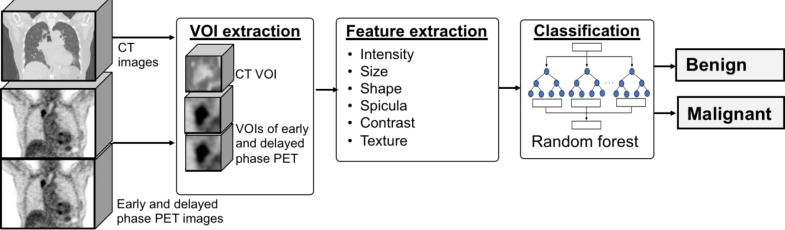
Outline of the proposed method

Figure 1 depicts an overview of the proposed method. In this method, regions designated as suspicious in the PET and CT images by the doctor were analysed using several characteristic features, and then automatically classified as benign or malignant. 

***Volume of Interest (VOI) extraction***

The position and diameter of the nodule to be analysed using conventional CT and PET/CT images was specified by the physician. Accordingly, the segmentation of the volume of interest (VOI) around the pulmonary nodule was carried out on the CT and PET images for analysis. The centre coordinates of the VOIs extracted from the conventional CT and PET/CT images were manually set while checking the MPR images of the CT images. 

First, the trans-axial image with the largest nodule area was located, and its centre coordinates were specified manually. Then, the longest diameter in the image was set as diameter, *D*_xy_, in the x-y direction (trans-axial plane) of the nodule. Subsequently, while changing the slice position in the direction of the body axis, the range of the slice in which the nodule was present was obtained and set as *D*_z_. The VOI was extracted using the number of pixels on three sides, namely 2*D*_xy_, 2*D*_xy_, and 2*D*_z_, from the original image.

***Extraction of characteristic features***

***Checkpoints in malignancy diagnosis***

Table 1 shows the checkpoints for distinguishing between benign and malignant nodules (11, 12). The physicians created this scheme by referring to the pixel values, such as the uptake value of the PET images and CT values. Furthermore, consideration was given to such factors as nodule components (e.g., ground glass opacity [GGO] or solid), shapes (roundness), clarity of the nodule border, and spiculas. In this study, these points were quantified as characteristic features.

***Characteristic features***

***(i) Pixel intensities of PET and CT images***

Many malignant nodules have a high pixel intensity in PET and CT images. Therefore, standardised uptake value (SUV) (13) of early and delayed PET images was defined as *ESUV* and *DSUV, *respectively. Furthermore, the difference in SUV between the delayed and early phases was defined as *ΔSUV*. In the measurement of SUV, two methods were introduced, namely SUV_max_ (hottest voxel) and SUV_peak_ (maximum average SUV within a 1 cm^3^ spherical volume). In the CT images, CT value at the centre of the nodule (*CT*_centre_) and the maximum CT value inside the nodule (*CT*_max_) were calculated.

***(ii) Shape***

With regard to the shape of the nodules, malignant nodules often have a ball-like shape, while the benign ones have a line-like shape. To evaluate the ball-like and line-like shapes, a method using a Hessian matrix was proposed (14). The Hessian matrix was obtained by taking the second order differential of the three-dimensional image as follows:

 (1) $$H=\left[\begin{array}{ccc} F_{\mathrm{xx}} & F_{\mathrm{xy}} & F_{\mathrm{xy}}\\ F_{\mathrm{xy}} & F_{\mathrm{yy}} & F_{\mathrm{yz}}\\ F_{\mathrm{zz}} & F_{\mathrm{zy}} & F_{\mathrm{zz}} \end{array}\right]$$

Then, three eigenvalues (λ_1_, e_2_, e_3_) were obtained from the matrix. Finally, the ball-like and line-like features (*L*_mass_ and *L*_line_) were calculated using the eigenvalues as follows: 

$L_{\mathrm{mass}}=\left|\lambda_{3}\right|/\lambda_{1}$

(2)

$L_{\mathrm{line}}=\left|\lambda_{2}\right|(\lambda_{2}-\lambda_{3})/\left|\lambda_{2}\right|$

(3)

***(iii) Contrast of the nodule border***

**Figure 2:**
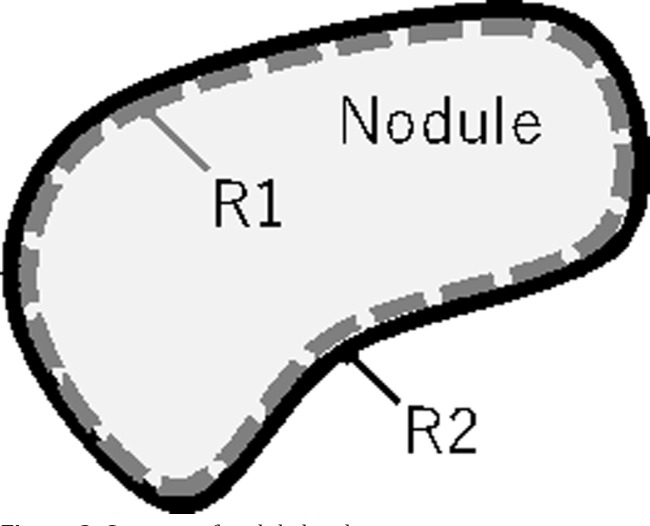
Contrast of nodule border

The border of a malignant nodule is often unclear. Therefore, the contrast of the border was evaluated using the difference between the CT values of the outer and inner borderlines of the nodules. In order to calculate this value, the average CT values at the pixels belonging to the inner edge R1 (*CT*_R1_) and the peripheral region R2 (*CT*_R2_) were obtained, and the difference between the two values, |*CT*_R1_-*CT*_R2_|, was defined as the contrast, *C*_b _(Figure 2). 

To obtain R1 and R2, the image was first binarized, and the contour was extracted by the Sobel operator. The set of pixels on the outline was defined as R2. Subsequently, the binarized region was shrunk by a morphological operation (erosion) with a structural element having a radius of 1 pixel, and the contour of the reduced region was extracted in the same manner as described above; a set of these pixels was used as R1.

***(iv) Spicula***

**Figure 3:**
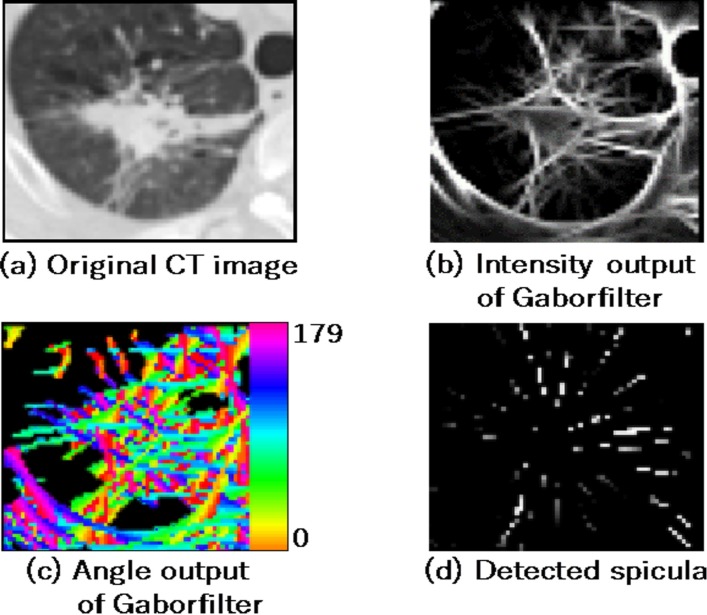
Detection of spicula

The presence of a spicula around the nodule increases the possibility of the nodule malignancy. In this study, spicula in CT images was detected using Gabor filter (15, 16). The use of Gabor filter facilitates the visualization of line patterns and their orientations (Figure 3b and 3c). Radial line patterns were extracted from the two images (Figure 3d), and the number of radial components and their ratios were calculated as the features of spiculas, *SP*_1_ and *SP*_2_.

***(v) Texture features***

The texture pattern of the lung lesion is important for the evaluation of malignancy. Out of the several ways to analyse textures, a method that is proposed by Haralick et al. based on the grey level co-occurrence matrix (GLCM) was employed in the current study (17). The matrix element P(i,j) of GLCM is the set of second order statistical probability values for changes between grey levels of *i* and *j* at a particular distance, *d*, and angle, *θ*. In this regard, *θ* represents the counter-clockwise angle with respect to the X axis.

The GLCM can assess such properties as texture uniformity, directionality, and contrast based on the distribution of the values of the matrix elements. Haralick et al. proposed 14 kinds of characteristic features using GLCM. In our study, it was necessary to limit the number of characteristic features as the number of the analysed cases was small. To obtain the texture features in each direction, the following five types of features were calculated using θ of 0° (*T*_1_0_ - *T*_5_0_) and 90° (*T*_1_90_ - *T*_5_90_) in the trans-axial plane of the CT images:

Contrast

$T_{1}=\sum_{i}\sum_{j}p\left(i,j\right){\left|i-j\right|}^{2}$

 (4)

Dissimilarity

$T_{2}=\sum_{i}\sum_{j}p\left(i,j\right)\left|i-j\right|$

 (5)

Correlation

$T_{3}=\sum_{i}\sum_{j}p\left(i-\mu_{i},j\right)(i-\mu_{i})P(i,j)/\sigma_{i}\sigma_{j}$

(6)

where, 

$\mu_{i}=\sum_{i}\sum_{j}\mathrm{ip}\left(i,j\right),\mu_{i}=\sum_{i}\sum_{j}\mathrm{jp}(i,j)$

(7)

$\sigma_{i}=\sqrt[{2}]{\sum_{i}\sum_{j}p(i,j){\left(i-\mu_{i}\right)}^{2}},\sigma_{i}=\sqrt[{2}]{\sum_{i}\sum_{j}p(i,j){\left(i-\mu_{i}\right)}^{2}}$

(8)

Homogeneity

$T_{4}=\sum_{i}\sum_{j}p\left(i,j\right)/(1+\left|i-j\right|)$

(9)

Energy

$\sigma_{i}=\sqrt[{2}]{\sum_{i}\sum_{j}p{\left(i,j\right)}^{2}}$

(10)

***Classification***

Identification of benign or malignant nodules was accomplished by means of the obtained characteristic features. In this study, classification was performed using the random forest algorithm (18). Random forest is an ensemble learning method for classification and regression that operates by constructing multiple decision trees and outputting the class that is the mode of the classes of the individual trees. Practically, the input for the random forest was the 25 characteristic values, while the output is a judgment result regarding the benignity or malignancy of the nodule. In this study, the maximum number of trees was set at 20. 

In order to analyse the distinguishing characteristics of this method, three kinds of classification methods were evaluated. These methods included: 1) classification based on CT images alone, 2) classification based on CT images and early phase PET images, and 3) classification based on CT images, as well as early and delayed phase PET images.

## Results

***Characteristic features***

In order to confirm whether individual feature values are useful for distinguishing between benign and malignant nodules, the mean, median, and standard deviation of the values in the two groups were calculated. 

The effect sizes (19) were also calculated. Furthermore, t-values and p-values were also calculated using the t-test (double-sided test). The results are shown in table 2.

**Table 1:** Checkpoints of benign and malignant nodules features

	**FDG uptake**	**CT value**	**Component**	**Shape**	**Border**	**Spicula**
**Benignity**	Low	Low	GGO	Line-like	Clear	Few
**Malignancy**	High Increase at delayed phase	High	Solid	Ball-like	Unclear	Many

CT: computed tomography, GGO: ground glass opacity, FDG: fluorodeoxyglucose

**Table 2:** Results of basic statistics and t-test

**Feature**	**Benign**			**Malignant**			**t-value**	**p-value**	**Effect size**
	**Mean**	**Median**	**SD**	**Mean**	**Median**	**SD**			
ESUV_max_	3.31	2.28	2.27	11.66	9.95	6.58	5.242	<0.001	3.685
DSUV_max_	3.81	2.37	3.08	14.29	11.76	8.3	5.165	<0.001	3.395
ESUV_peak_	2.31	1.69	1.42	7.66	5.49	4.78	4.684	<0.001	3.764
DSUV_peak_	2.51	1.56	1.79	9.46	6.83	6.29	4.638	<0.001	3.886
ΔSUV_max_	0.5	0.21	0.88	2.63	1.96	1.87	4.49	<0.001	2.4
ΔSUV_peak_	0.2	0.05	0.44	1.8	1.05	2.04	3.348	0.003	3.663
T_4-0_	0.0651	0.0602	0.0239	0.045	0.0418	0.0117	3.289	0.003	0.839
T_4-90_	0.0655	0.0614	0.0234	0.0459	0.0437	0.0114	3.274	0.003	0.834
T_5-0_	0.0345	0.0305	0.0192	0.0209	0.0184	0.0084	2.837	0.009	0.708
D_xy_	17.2	17.5	3.8	22.1	22.8	6.7	2.771	0.01	1.279
T_5-90_	0.0342	0.0301	0.0179	0.0211	0.0193	0.008	2.92	0.08	0.732
C_b_	0.0811	0.0844	0.0305	0.0941	0.0964	0.0137	1.692	0.104	0.425
T_2-0_	33.53	33.9	8.27	36.8	37.21	3.09	1.615	0.121	0.394
L_mass_	56	22.6	104.4	193.2	47.1	365.9	1.574	0.131	1.313
CT_max_	983.9	661.3	710.7	727.3	535.5	448.8	1.333	0.193	0.361
T_3-0_	0.63	0.668	0.154	0.575	0.576	0.104	1.31	0.2	0.362
T_2-90_	34.36	32.58	8.63	37.02	35.3	4.408	1.198	0.242	0.308
T_1-0_	2616.2	2536.8	1096	2885.4	2855.9	395.9	1.009	0.324	0.245
D_z_	18.1	16.5	7.9	20.6	19.5	8.1	0.955	0.346	0.312
CT_centre_	0.3	19.9	151	26.1	35.9	34.1	0.729	0.475	0.171
T_3-90_	0.612	0.674	0.187	0.58	0.604	0.093	0.671	0.508	0.171
T_1-90_	2734.4	2500.2	1205.3	2916.3	2761.6	619.5	0.586	0.563	0.15
SP_2_	0.0195	0.0216	0.0104	0.0147	0.0184	0.0161	0.359	0.722	0.101
SP_1_	73.7	42	103.5	82.7	75.5	52.8	0.338	0.738	0.086
L_line_	42.5	24.4	57.8	43.4	28.9	33.6	0.061	0.952	0.016

ESUV_max_: maximum standardised uptake value of early PET images, DSUV_max: _maximum standardised uptake value of delayed PET images, ΔSUV_max: _difference between ESUV_max _and DSUV_max_, ESUV_peak_: peak standardised uptake value of early PET images, DSUV_peak_: peak standardised uptake value of delayed PET images, ΔSUV_peak_: difference between ESUV_peak_ and DSUV_peak_, CT_centre_: CT value at the centre of the nodule, CT_max_: maximum CT value inside the nodule, D_xy _and D_z_: nodule diameters in X-Y plane (trans-axial) and z-direction, *L*_mass_: ball-like feature, L_line_: line-like feature,* C*_b_:* CT*_R1_-*CT*_R2_, *SP*_1_ and *SP*_2_: features of spicula, *T*_1_0_~*T*_5_0_: texture features for horizontal direction, *T*_1_90_~*T*_5_90_: texture features for vertical direction

CT: computed tomography, PET: positron emission tomography

**Figure 4:**
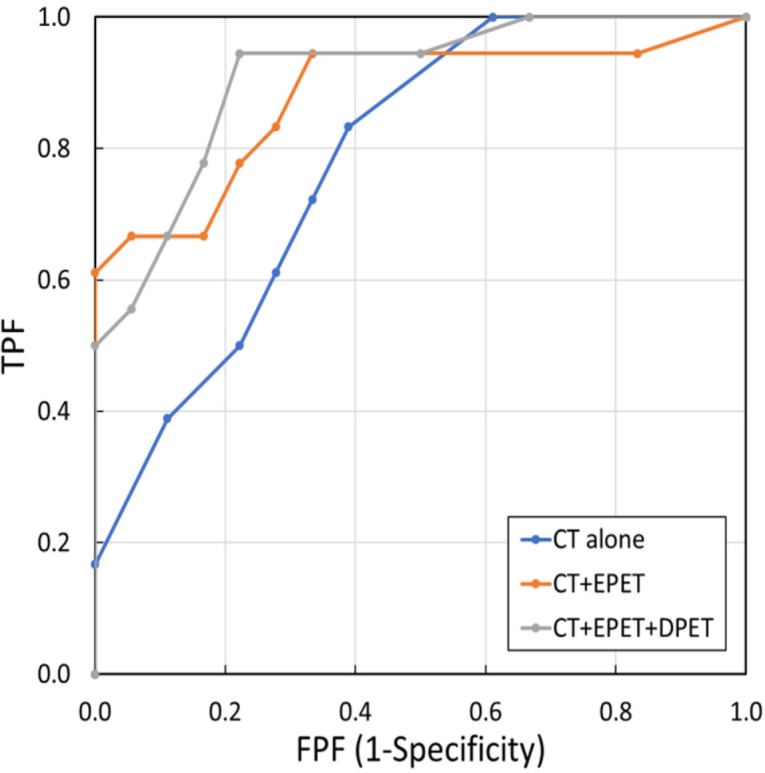
Receiver operating characteristic (ROC) curves of classification

CT: computed tomography, DPET: delayed phase positron emission tomography, EPET: early phase positron emission tomography, FPF: false-positive fraction, ROC: receiver operating characteristics, TPF: true-positive fraction

**Figure 5:**
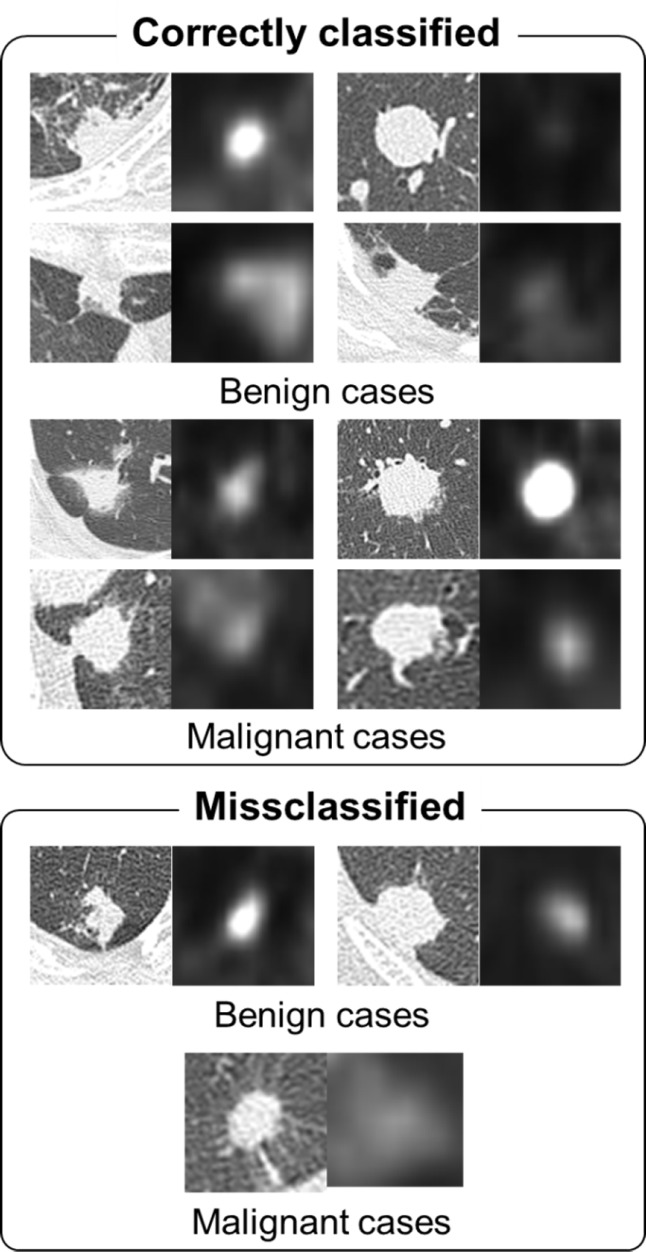
classification results(Images on the left are CT images and images on the right are early phase PET images. The two values under the images are the SUVs of early and delayed PET images).

CT: computed tomography, PET: positron emission tomography, SUV: standardised uptake values

There was a significant difference between the characteristic features of benign and malignant nodules with regard to SUV. The PET examination resulted in the most significant SUV that was confirmed to be useful for distinguishing between benignity and malignancy. In addition, some texture features showed significant differences between benign and malignant nodules and were effective for distinguishing between these two states. However, difference in features with regard to spicula was of low significance. Furthermore, the average CT_max_ in the benign nodules was higher than that in the malignant nodules.

***Results of classification scheme***

The nodule classification scheme was evaluated using the receiver operating characteristic (ROC) curve. In the curve, the true positive rate was defined as the ratio of the number of corrected malignant nodules to the total number of malignant nodules. On the other hand, false positive rate was defined as the ratio of the number of misclassified benign nodules to the total number of benign nodules. Furthermore, performance was evaluated by the leave-one-out cross-validation method.

To analyse the distinguishing characteristics of our technique, three methods were evaluated. These methods included: 1) classification based on CT images alone, 2) classification based on CT images and early phase PET images, and 3) classification based on CT images, as well as early and delayed phase PET images. Figure 4 displays the ROC curves of each of the above methods. The area under the curves (AUC) of methods 1, 2, 3 were 0.730, 0.860, and 0.895, respectively. 

Considering the accuracy rate of malignant nodules (0.944), the accuracy rates of benign nodules for methods 1, 2, and 3 were obtained as 0.277, 0.611, and 0.722, respectively. The CT and PET images in trans-axial plane with the accuracy rates of 0.722 and 0.944 for the benign and malignant nodules, respectively are shown in Figure 5. There were significant differences between ROC curves 1 and 2, 2 and 3, as well as 1 and 3 (P=0.032, P=0.104, and P=0.021, respectively).

## Discussion

The findings of the present study revealed a significant difference between the characteristic features of benign and malignant nodules with regard to SUV (Table 2). The SUV obtained by PET examination was confirmed to be useful for distinguishing between benignity and malignancy. However, given the fact that inflammatory diseases can also result in high SUVs, these values should be combined with other features for correct classification. In addition, some texture features were effective for distinguishing between benign and malignant nodules. 

Difference in features with regard to spicula was of low significance. This is because many spiculas are observed even in inflammatory diseases. Furthermore, the average CT_max_ in the benign nodules was higher than that in the malignant ones. This is due to the presence of calcification inside the benign nodules. The overlap of the characteristic features between two classes makes it difficult to distinguish between them using a single feature. Therefore, the integration of multiple characteristic features using the classifier would be more effective. 

In the ROC curves (Figure 4), the proposed method based on the combined use of CT and two-phase PET images showed an incorrect detection rate of 0.278 for the benign nodules (accuracy rate: 0.722), whereas the accuracy rate of detecting a malignant nodule was 0.944. Target nodules in this study were “*difficult nodules*” to differentiate based on CT and PET/CT images. Most of the benign cases were not confirmed in follow-up examinations; however, they were confirmed after biopsy. Our results indicated that biopsy examination with its accompanying physical hardship to the patient, especially in benign cases, could be reduced by 72.2%.

As shown in Figure 4, the use of PET plus CT images improves the AUC of the ROC curves, compared to the employment of only CT images. This denotes the effectiveness of using both anatomical and functional information together. Based on the ROC curves, there was a significant difference between the analysis using CT alone and the one using both CT and PET images. In terms of the analysis that was based on PET plus CT images, no significant difference was found between the analysis made based on early phase alone and the one conducted using the early and delayed phases together. However, false-positive rate at the high true positive rate is very important in malignancy analysis. Based on the above-mentioned results, analysis using two-phase images was still advantageous.

Even with the use of this method, there were some nodules that were misclassified. One of the reasons for this is that the CT and PET images did not show the characteristics that were peculiar to benign or malignant lesions. The future challenge is to introduce the novel characteristic features that reflect benignity and malignancy. Moreover, the further development of this method requires the improvement of the classifiers’ performance. Therefore, it is necessary to compare the performance of this method with classifiers, such as linear discriminant analysis (LDA), quadratic discriminant analysis (QDA), support vector machine (SVM), and artificial neural network (ANN).

There are many reports indicating that machine-learning performances are remarkably improved by using deep learning (20, 21). In earlier studies, we applied the deep learning technique for automated nodule detection and classification of lung cancer types (22, 23). In the future, we intend to apply the deep learning technique for the classification of pulmonary nodules.

## Conclusion

In this study, we have developed a machine learning-based analysis of pulmonary nodules using early and delayed phase PET and conventional CT images in patients undergoing biopsy. As a result, 94.4% of the malignant nodules were identified correctly assuming that 72.2% of the benign nodules were judged correctly. These results indicate that the proposed method may be useful to improve the accuracy of malignancy analysis. 
